# Exploring FlyBase Data Using QuickSearch

**DOI:** 10.1002/cpz1.731

**Published:** 2023-04-04

**Authors:** Steven J. Marygold, the FlyBase Consortium

**Affiliations:** ^1^ Department of Physiology, Development and Neuroscience University of Cambridge Downing Street Cambridge United Kingdom

**Keywords:** biological database, *Drosophila melanogaster*, FlyBase, query tools, QuickSearch

## Abstract

FlyBase (www.flybase.org) is the primary online database of genetic, genomic, and functional information about *Drosophila melanogaster*. The long and rich history of *Drosophila* research, combined with recent surges in genomic‐scale and high‐throughput technologies, means that FlyBase now houses a huge quantity of data. Researchers need to be able to query these data rapidly and intuitively, and the QuickSearch tool has been designed to meet these needs. This tool is conveniently located on the FlyBase homepage and is organized into a series of simple tabbed interfaces that cover the major data and annotation classes within the database. This article describes the functionality of all aspects of the QuickSearch tool. With this knowledge, FlyBase users will be equipped to take full advantage of all QuickSearch features and thereby gain improved access to data relevant to their research. © 2023 The Authors. Current Protocols published by Wiley Periodicals LLC.

**Basic Protocol 1**: Using the “Search FlyBase” tab of QuickSearch

**Basic Protocol 2**: Using the “Data Class” tab of QuickSearch

**Basic Protocol 3**: Using the “References” tab of QuickSearch

**Basic Protocol 4**: Using the “Gene Groups” tab of QuickSearch

**Basic Protocol 5**: Using the “Pathways” tab of QuickSearch

**Basic Protocol 6**: Using the “GO” tab of QuickSearch

**Basic Protocol 7**: Using the “Protein Domains” tab of QuickSearch

**Basic Protocol 8**: Using the “Expression” tab of QuickSearch

**Basic Protocol 9**: Using the “GAL4 etc” tab of QuickSearch

**Basic Protocol 10**: Using the “Phenotype” tab of QuickSearch

**Basic Protocol 11**: Using the “Human Disease” tab of QuickSearch

**Basic Protocol 12**: Using the “Homologs” tab of QuickSearch

**Support Protocol 1**: Managing FlyBase hit lists

## INTRODUCTION

FlyBase (www.flybase.org) is the primary online database of genetic, genomic, and functional information about the model organism *Drosophila melanogaster*. Data are incorporated through curation of published research papers and via computational uploads of third‐party datasets. Much of these data is partitioned across ∼20 “data classes” within FlyBase (Jenkins et al., 2022; Table 1). These include classical divisions, such as “genes,” “alleles,” or “references,” as well as more recent additions such as “sequence features,” “human disease models,” or “gene groups.” Certain data classes are associated with functional, phenotypic, or expression annotations, which themselves use terms from structured and controlled vocabularies (ontologies; Table 2). Links are made between all related objects. For example, a phenotype ontology term may be associated with a particular allele of a specified gene, all of which will be attributed to the source reference. This organization underlies the presentation of related data within distinct report pages within FlyBase and enables users to browse, navigate, and search effectively across the website.

**Table 1:** Major Data Classes in FlyBase and How They Can be Queried Using QuickSearch

Data Class	Searchable via these QuickSearch tabs
***Genetic/genomic data***	
Aberrations	Search FlyBase, Data Class: Aberration
Alleles	Search FlyBase, Data Class: Allele
Balancers	Search FlyBase, Data Class: Balancer
Genes	Search FlyBase, Data Class: Gene
Insertions	Search FlyBase, Data Class: Insertion
Natural transposons	Search FlyBase, Data Class: Natural Transposon
Orthologs/paralogs	Homologs
Physical interactions	Search FlyBase, Data Class: Physical Interaction
Polypeptides	Search FlyBase, Data Class: Polypeptide
Sequence features	Search FlyBase, Data Class: Sequence Feature
Transcripts	Search FlyBase, Data Class: Transcript
Transgenic constructs	Search FlyBase, Data Class: Transgenic Construct
***Reagents***	
Cell lines	Search FlyBase, Data Class: Cell Line
Chemicals	Search FlyBase, Data Class: Chemical
Clones	Search FlyBase, Data Class: Clone
Experimental tools	Search FlyBase, Data Class: Experimental Tool
Stocks	Search FlyBase, Data Class: Stock
Strains	Search FlyBase, Data Class: Strain
***Integrated data***	
Datasets	Search FlyBase, Data Class: Dataset
Gene groups	Search FlyBase, Gene Groups, Data Class: Gene Group
Human disease models	Search FlyBase, Human Disease, Data Class: Human Disease Model
Pathways	Search FlyBase, Pathways, Data Class: Gene Group
***Other***	
Images	Search FlyBase, Data Class: Image
References	Search FlyBase, References, Data Class: Reference

**Table 2:** Ontologies Used in FlyBase, Searchable via the “Search FlyBase” or “Data Class” Tabs of QuickSearch

Ontology	Used to annotate
Anatomy Ontology	Phenotypes, genetic interactions, expression
Development Ontology	Phenotypes, genetic interactions, expression
Disease Ontology	Disease models
FlyBase Ontology	Phenotypic class, mutagens, allele class, publication types
Gene Ontology	Function of genes and gene groups
Image Method Ontology	Images
Molecular Interaction Ontology	Physical interactions
Sequence Ontology	Sequence features and variants
Stock Ontology	Stock descriptions/collections

The QuickSearch tool in FlyBase provides rapid querying of all these data classes, annotations, and ontologies through a series of concise tabbed interfaces (Table 3). QuickSearch is accessible directly from the homepage and is designed to provide quick and intuitive access to searches that users commonly need to perform. The “Search FlyBase” tab of QuickSearch allows for Google‐like querying across all data classes, whereas the other tabs permit more targeted queries of particular data classes (References, Gene Groups, Pathways, Human Disease, Homologs, and Data Class), ontologies used during annotation [Gene Ontology (GO), Disease Ontology (DO)], or annotations associated with one or more these data classes (Expression, Phenotype, GAL4, etc.).

**Figure 1:**
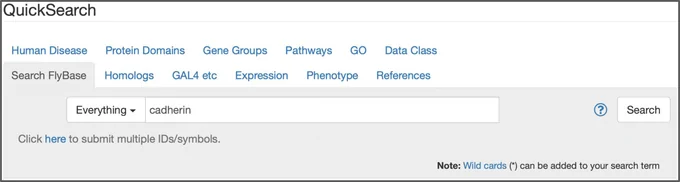
The “Search FlyBase” tab of QuickSearch, with “Everything” selected as the search mode and the text “cadherin” entered as the search term.

**Table 3:** Summary of QuickSearch Tabs and Their Functionality

QuickSearch tab	Search domain	Primary entity retrieved
Search FlyBase	All of database	All data classes and ontologies
Data Class	Single data class/ontology	Specified data class/ontology
References	Reference data class	References
Gene Groups	Gene Group data class	Gene groups
Pathways	Pathway data class	Pathways
GO	Gene ontology	GO terms
Protein Domains	Protein domain annotations	Genes
Expression	Expression annotations	Genes
GAL4 etc	Expression annotations	Alleles and transgenic constructs/insertions
Phenotype	Phenotype annotations	Alleles
Human Disease	Human Disease Model data class, disease ontology (DO), DO annotations, and OMIM associations	Human disease models, DO terms, genes/alleles associated with those models/terms
Homologs	Orthologs and paralogs	Genes

The basic protocols in this article cover the functionality and usage of each QuickSearch tab in turn. The supporting protocol details effective management and processing of the hit lists generated from most QuickSearch queries. The concluding commentary section describes critical parameters to consider for successful searching, together with advice on interpreting search results and troubleshooting any problems. The protocols and screenshots are based on FlyBase release FB2022_06 (December 2022).

## Basic Protocol 1 USING THE “SEARCH FlyBase” TAB OF QUICKSEARCH

This tab (Fig. 1) performs a comprehensive case‐insensitive search of all FlyBase data classes and ontologies. It provides a “Google‐like” functionality in that no prior knowledge of the content or organization of FlyBase data is required. The search box accepts any text, and all possible matches are retrieved and reported rapidly. The “Search FlyBase” tab is often the best place to start a search of data within FlyBase.

### Necessary Resources

#### Hardware

Computer or other device with access to the internet

##### Software

An up‐to‐date web browser, such as Firefox, Chrome, or Safari, with JavaScript and cookies enabled

1 Click on the Search FlyBase tab of QuickSearch (www.flybase.org).
2 Choose whether to restrict the search to “ID/Symbol/Name” (the default) or to search for matches in the text of all report fields by selecting “Everything” in the drop‐down menu.
  The “ID/Symbol/Name” option includes synonyms. Searching “Everything” may result in many more hits returned, but this option is useful if searching for a text string within a particular data class that is not an ID/symbol/name.
3 Enter one or more search terms.
  The search term box of the Search FlyBase tab supports several additional features that can be used to narrow or broaden the query. A wildcard character (*) can be appended, prepended, or included within a search term to broaden the query. When specifying multiple terms, a Boolean “AND” is used for searches by default and does not require any special notation. A Boolean “OR” can be added to find records that have one or another of a list of specified terms. Results can be specified to contain an exact phrase by surrounding the search term with double quotes.
4 Click the “Search” button or press “enter.”
5 View the hit list of results matching the search criteria.
  Results are listed in descending order of their relevance score.
6 Click on an individual entry to view its report page or choose to perform another action on the hit list (see Support Protocol 1).
  FlyBase report pages display all data associated with that entity, organized into separate sections. Reports contain many links to other related reports within and external to FlyBase to aid further navigation and exploration.

## Basic Protocol 2 USING THE “DATA CLASS” TAB OF QuickSearch

**Figure 2:**
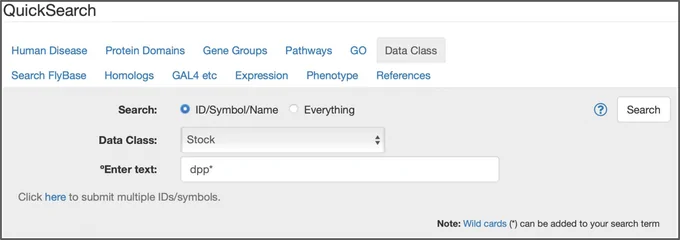
The “Data Class” tab of QuickSearch, with “ID/Symbol/Name” selected as the search mode, “stock” selected from the Data Class menu, and “dpp*” entered as the search term.

If a query is well defined, it is useful to perform a more targeted search than that offered by the Search FlyBase tab to reduce/prevent false positive results. This can be achieved using the “Data Class” tab (Fig. 2) where queries can be restricted to a single class of data (Table 1) or ontology (Table 2). This functionality is particularly useful for querying specific data classes/ontologies that do not have a dedicated QuickSearch tab.

### Necessary Resources

#### Hardware

Computer or other device with access to the internet

##### Software

An up‐to‐date web browser, such as Firefox, Chrome, or Safari, with JavaScript and cookies enabled

1 Click on the Data Class tab of QuickSearch (www.flybase.org).
2 Select the desired data class or ontology from the drop‐down menu.
  Data class options are shown in Table 1 and ontology options are shown in Table 2. Alternatively, the “All data types” option may be selected, though doing so simply reproduces the functionality of the “Search FlyBase” tab.
3 Choose whether to restrict the search to “ID/Symbol/Name” (the default) or to search for matches in the text of all report fields by clicking the “Everything” button.
  The “ID/Symbol/Name” option includes synonyms. Searching “Everything” may result in many more hits returned, but this option is useful if searching for a text string within a particular data class that is not an ID/symbol/name.
4 Enter one or more search terms appropriate to the selected class/ontology.
  The search term box of the Search FlyBase tab supports several additional features that can be used to narrow or broaden the query. A wildcard character (*) can be appended, prepended, or included within a search term to broaden the query. When specifying multiple terms, a Boolean “AND” is used for searches by default and does not require any special notation. A Boolean “OR” can be added to find records that have one or another of a list of specified terms. Results can be specified to contain an exact phrase by surrounding the search term with double quotes.
  Valid entries are ID/Symbol/Name (or their synonyms), if that option has been selected; valid entry symbols will start to appear when typing and can be clicked to populate the field. The search is case‐insensitive and a wildcard (*) can be added to match partial terms.
5 Click the “Search” button or press “enter.”
6 View the hit list of results matching the search criteria.
  Results are listed in descending order of their relevance score.
7 Click on an individual entry to view its report page or choose to perform another action on the hit list (see Support Protocol 1).
  FlyBase report pages display all data associated with that entity, organized into separate sections. Reports contain many links to other related reports within and external to FlyBase to aid further navigation and exploration.

## Basic Protocol 3 USING THE “REFERENCES” TAB OF QuickSearch

**Figure 3:**
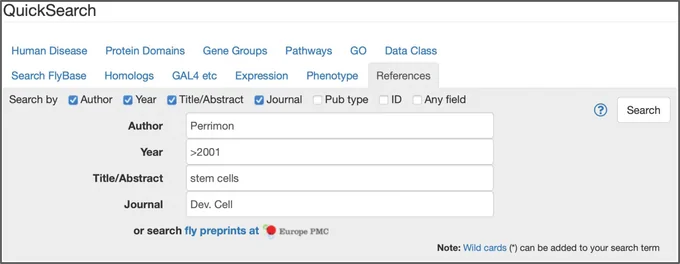
The “References” tab of QuickSearch, with several criteria entered as search terms.

FlyBase maintains a large and varied bibliography of *Drosophila*‐specific publications (Marygold et al., 2013). It currently comprises >237,000 references, covering ∼40 distinct publication types, with dates of publication extending from the 17th century to the present day. Approximately half of these references are primary research papers, identified through regular searches of the NCBI PubMed database. Other publication types include reviews, commentaries, and errata (also identified in PubMed), as well as otherwise unpublished “personal communications” from the *Drosophila* community and internal “FlyBase analyses.” The entire FlyBase bibliography is searchable using the “References” tab (Fig. 3).

### Necessary Resources

#### Hardware

Computer or other device with access to the internet

##### Software

An up‐to‐date web browser, such as Firefox, Chrome, or Safari, with JavaScript and cookies enabled

1 Click on the References tab of QuickSearch (www.flybase.org).
2 Add search terms using the default “Any field” option or click the appropriate box to use additional, more specific search fields.
  Additional search fields are “Author,” “Year,” “Title/Abstract,” “Journal,” “Publication type,” and “ID” [i.e., PubMed ID, PubMed Central ID, Digital Object Identifier (DOI), and FlyBase reference ID (FBrf)]. Alternatively, click on the link to “search fly preprints at Europe PMC,” as preprints are not currently indexed or curated at FlyBase.
3 Enter one or more terms that are appropriate to the selected field.
  Valid entry terms will start to appear in the “Author,” “Journal,” and “Publication type” fields when typing and can be clicked to populate the field. Searches are case‐insensitive and a wildcard (*) can be added to match partial terms. Boolean operators (AND, OR, BUT, and NOT) can be used within any field to search using specific combinations or exclusions. The “Year(s)” field also accepts ranges and greater/less than notations (e.g., “2004‐2008” or “>2001”).
4 Click the “Search” button or press “enter.”
5 View the hit list of matching references.
  Results are listed in descending order of their relevance score.
6 Click on an individual reference to view its Reference Report or choose to perform another action on the hit list (see Support Protocol 1).
  Reference Reports display the full citation data of the reference, together with links to the journal article at the publisher's site, the PubMed record, and (where available) the full text at PubMed Central and Europe PubMed Central. In addition, Reference Reports include a summary of the data associated with the publication in FlyBase, and each associated entity is linked to its own dedicated report.

## Basic Protocol 4 USING THE “GENE GROUPS” TAB OF QuickSearch

**Figure 4:**
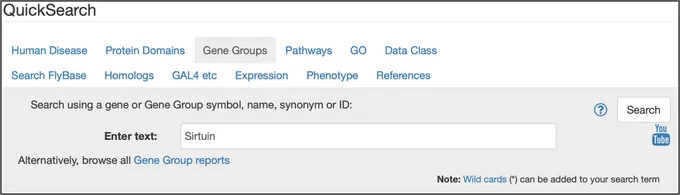
The “Gene Groups” tab of QuickSearch, with the text “Sirtuin” entered as the search term. Note the link to “browse all Gene Group reports.”

The Gene Groups resource in FlyBase comprises manually compiled sets of *D. melanogaster* genes that share a set of characteristics (Attrill et al., 2016). Examples include evolutionarily related gene families (e.g., actins and odorant receptors), subunits of macromolecular complexes (e.g., spliceosomes and ribosomes), and gene products with a common molecular function (e.g., deubiquitinases and GTPase activating proteins). These collections are useful starting points for further analyses and explorations of associated data, both within FlyBase and in other species databases. All groups are searchable and browsable via the “Gene Groups” tab (Fig. 4).

### Necessary Resources

#### Hardware

Computer or other device with access to the internet

##### Software

An up‐to‐date web browser, such as Firefox, Chrome, or Safari, with JavaScript and cookies enabled

1 Click on the Gene Groups tab of QuickSearch (www.flybase.org).
2 Enter a name, a symbol, or any other identifier corresponding to a group or a gene member.
  Valid entries are symbols or full names (or their synonyms), FlyBase identifiers for the Gene Groups, or any member genes. Valid names of Gene Groups will start to appear when typing and can be clicked to populate the field. The search is case‐insensitive and a wildcard (*) can be added to match partial terms.
  Alternatively, click on the “browse” link to see a page indexing all Gene Group Reports.
3 Click the “Search” button or press “enter.”
4 View the hit list of matching gene groups.
  Results are listed in descending order of their relevance score.
5 Click on an individual group to view its Gene Group Report or choose to perform another action on the hit list (see Support Protocol 1).
  Gene Group Reports include a brief description of the group, a summary of the references used to compile the group, the key GO terms that typify the members, and links to related Gene Groups within FlyBase and at external sites [e.g., human gene families at the HUGO Gene Nomenclature Committee (HGNC) website] (Seal et al., 2023). The “Members” table lists all members of the group with links to the corresponding Gene Report pages, together with convenient buttons to export members to other tools, such as a hit list or Batch Download, to facilitate further analyses.

## Basic Protocol 5 USING THE “PATHWAYS” TAB OF QuickSearch

**Figure 5:**
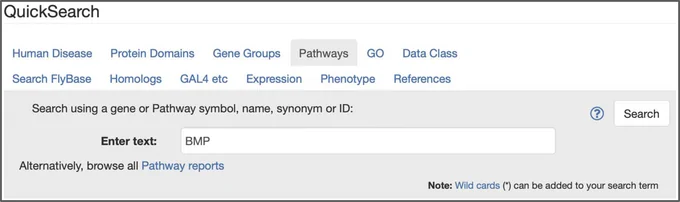
The “Pathways” tab of QuickSearch, with the text “BMP” entered as the search term. Note the link to “browse all Pathway reports.”

The Pathways resource in FlyBase is a sub‐category of the Gene Group resource and comprises sets of *D. melanogaster* genes whose products have been experimentally shown to act within or regulate a signaling pathway (Larkin et al., 2021). All Pathway Reports are compiled manually by FlyBase curators based on the research literature, using standards for inclusion agreed by the GO consortium (The Gene Ontology Consortium, 2021). They are searchable and browsable via the “Pathways” tab (Fig. 5).

### Necessary Resources

#### Hardware

Computer or other device with access to the internet

##### Software

An up‐to‐date web browser, such as Firefox, Chrome, or Safari, with JavaScript and cookies enabled

1 Click on the Pathways tab of QuickSearch (www.flybase.org).
2 Enter a name, a symbol, or any other identifier corresponding to a pathway or a pathway member.
  Valid entries are symbols or full names (or their synonyms), FlyBase identifiers for the Pathways, or any member genes. Valid names of curated Pathways will start to appear when typing and can be clicked to populate the field. The search is case‐insensitive and a wildcard (*) can be added to match partial terms.
  Alternatively, click on the “browse” link to see a page indexing all Pathway Reports.
3 Click the “Search” button or press “enter.”
4 View the hit list of matching pathways.
  Results are listed in descending order of their relevance score.
5 Click on an individual pathway to view its Pathway Report or choose to perform another action on the hit list (see Support Protocol 1).
  Pathway Reports include a brief description and thumbnail image of the pathway, selected references for background information, corresponding GO Biological Process term, physical interaction network of pathway members, and links to related Gene Groups within FlyBase and at external sites. The interactive “Members” table lists all members of the pathway alongside other relevant information, together with convenient buttons to export members to other tools, such as a hit list or Batch Download, to facilitate further analyses.

## Basic Protocol 6 USING THE “GO” TAB OF QuickSearch

**Figure 6:**
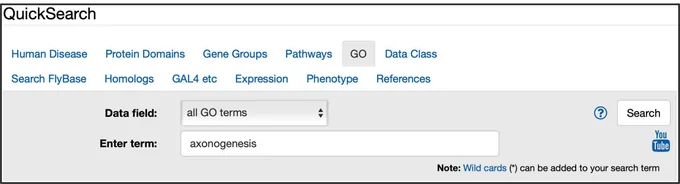
The “GO” tab of QuickSearch, with the “Data field” set to search “all GO terms” and the text “axonogenesis” entered as the search term.

The GO is used in FlyBase and many other biological databases to describe the attributes of gene products in terms of their molecular functions, associated biological processes, and cellular components (The Gene Ontology Consortium, 2021; Tweedie et al., 2009). The GO tab (Fig. 6) can be used to find terms of interest within the GO and then identify *Drosophila* genes (or gene groups) annotated with those terms.

### Necessary Resources

#### Hardware

Computer or other device with access to the internet

##### Software

An up‐to‐date web browser, such as Firefox, Chrome, or Safari, with JavaScript and cookies enabled

1 Click on the GO tab of QuickSearch (www.flybase.org).
2 Choose to search “all GO terms” (which is the default) or to restrict the search to the individual “molecular function,” “biological process,” or “cellular component” ontologies by using the “Data field” drop‐down menu.
3 Enter the search text or identifier.
  Valid entries are GO terms/synonyms or GO identifiers. Valid GO terms that match the entered text, confined to the ontology aspect selected in step 2, will start to appear when typing and can be clicked to populate the field. The search is case‐insensitive and a wildcard (*) can be added to match partial terms.
4 Click the “Search” button or press “enter.”
5 View the hit list of matching GO terms.
6 Click on the desired GO term to view its Term Report page.
  These reports include a definition, synonyms, and a view of the tree displaying the hierarchical relationships between any parent and child terms. Within the tree view, the total number of records annotated with each GO term (including its children) is indicated in green text. Link‐outs to the corresponding term page at the QuickGO (Binns et al., 2009) and AmiGO (Carbon et al., 2009) websites are also provided.
7 If present, click the “Genes” (or “Gene Groups”) button within the “Annotations” section to view a hit list of genes (or gene groups) annotated with the given term or any of its child terms.
8 Click on an individual gene (or gene group) to view its report page and associated GO annotations or choose to perform another action on the hit list (see Support Protocol 1).
  Gene Reports list individual GO annotations within the “Function” section, accompanied by a “ribbon” summarizing the major annotation categories for each of the Molecular Function, Biological Process, and Cellular Component aspects. Gene Group reports list the key GO terms applicable to the group within the “Description” section. A GO ribbon stack summarizing annotations for all member genes is shown in the “Members” section.

## Basic Protocol 7 USING THE “PROTEIN DOMAINS” TAB OF QuickSearch

**Figure 7:**
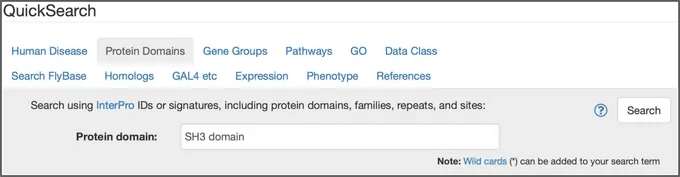
The “Protein Domains” tab of QuickSearch, with the text “SH3 domain” entered as the search term.

This tab (Fig. 7) is used to generate lists of *D. melanogaster* genes whose protein products have a specified domain or functional site, as determined by InterPro (Paysan‐Lafosse et al., 2023). InterPro integrates protein signatures from several individual sources (including Pfam and SMART) and then associates InterPro entries with protein products in the UniProtKB collection (The UniProt Consortium, 2021). UniProtKB IDs are linked to gene records in FlyBase, thereby providing the connection between InterPro protein features and *D. melanogaster* genes.

### Necessary Resources

#### Hardware

Computer or other device with access to the internet

##### Software

An up‐to‐date web browser, such as Firefox, Chrome, or Safari, with JavaScript and cookies enabled

1 Click on the Protein Domains tab of QuickSearch (www.flybase.org).
2 Enter a search term or an identifier.
  Valid entries are InterPro terms or identifiers for protein domains, families, homologous superfamilies, repeats, or sites. Valid entry terms/IDs will start to appear when typing and can be clicked to populate the field. The search is case‐insensitive and a wildcard (*) can be added to match partial terms.
3 Click the “Search” button or press “enter.”
4 View the hit list of genes whose product(s) are associated with the specified search term.
  Results are listed in descending order of their relevance score.
5 Click on an individual gene to view its Gene Report or choose to perform another action on the hit list (see Support Protocol 1).
  Protein domain information from InterPro is displayed within the “Function” and “Gene Models and Products” sections of a Gene Report. Click on an InterPro term to navigate to its report on the InterPro site, which contains additional information.
  Click on the JBrowse link in the Gene Report and choose to display the Pfam and/or SMART “Protein Domains” track(s) to view how these domains align with the intron/exon structure of the gene.

## Basic Protocol 8 USING THE “EXPRESSION” TAB OF QuickSearch

**Figure 8:**
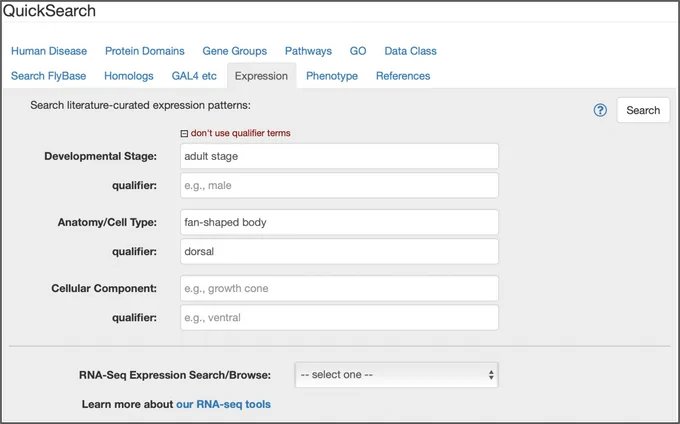
The “Expression” tab of QuickSearch, with several criteria entered as search terms.

This tab (Fig. 8) is used to search for *D. melanogaster* genes with a specified spatial and/or temporal expression pattern, as curated from low‐throughput studies of the transcript or polypeptide described in the literature. Expression data may be searched by developmental stage, anatomy/cell type, or subcellular location, or any combination of these. The lower panel of the Expression tab allows access to different options for querying or browsing high‐throughput RNA‐Seq expression data.

### Necessary Resources

#### Hardware

Computer or other device with access to the internet

##### Software

An up‐to‐date web browser, such as Firefox, Chrome, or Safari, with JavaScript and cookies enabled

1 Click on the Expression tab of QuickSearch (www.flybase.org).
2 Enter a term into the “Developmental Stage,” “Anatomy/Cell Type,” or “Cellular Component” field.
  Valid entries are terms or identifiers from the fly developmental stage, fly anatomy, or GO cellular component ontologies, respectively. Valid terms that match the entered text will start to appear when typing and can be clicked to populate the field. Note that the autocomplete function only lists terms for which annotations exist. The search is case‐sensitive; wildcards (*) are not accepted.
  Note that searches will return hits matching the selected ontology term or any of its child terms. For example, searching with “imaginal disc” will return genes annotated with that exact term as well as terms with an is_a relationship (e.g., wing disc and eye disc) or a part_of relationship (e.g., imaginal disc posterior compartment and wing pouch) to “imaginal disc.”
  Alternatively, click the “Learn more about our RNA‐Seq tools” link in the lower section to access different options for querying or browsing high‐throughput RNA‐Seq expression data.
3 If desired, click the “refine search by adding qualifier terms” text and enter a field‐appropriate “qualifier” term in the box that appears below the main search box.
  For example, the developmental stage can be qualified by specifying gender, whereas the anatomy and subcellular fields can be qualified by a term adding detail about spatial distribution (e.g., “dorsal”) or signal intensity (e.g., “faint”). Valid qualifier terms that match the entered text will start to appear when typing and can be clicked to populate the field; only those qualifiers associated with the term(s) chosen in step 2 will appear. The search is case‐sensitive; wildcards (*) are not accepted.
4 If desired, repeat steps 1 and 2 for the other search field(s).
  The autocomplete function is coordinated between the terms entered in the primary search boxes; only terms that have been co‐annotated with those already filled will be offered.
  It is not necessary to fill all three primary search boxes, but multiple fields may be completed in any combination to add additional constraints to a query.
5 Click the “Search” button or press “enter.”
6 View the hit list of genes that exhibit a transcript or protein expression pattern matching the search criteria.
  Results are listed in descending order of their relevance score.
7 Click on an individual gene to view its Gene Report or choose to perform another action on the hit list (see Support Protocol 1).
  Detailed expression information associated with the transcript or polypeptide, or that deduced from a reporter is included within the “Expression Data” section of the Gene Report. This section also displays expression data derived from high‐throughput studies and “ribbons” summarizing gene expression in different cell types, anatomical parts, and developmental stages.

## Basic Protocol 9 USING THE “GAL4 ETC” TAB OF QuickSearch

**Figure 9:**
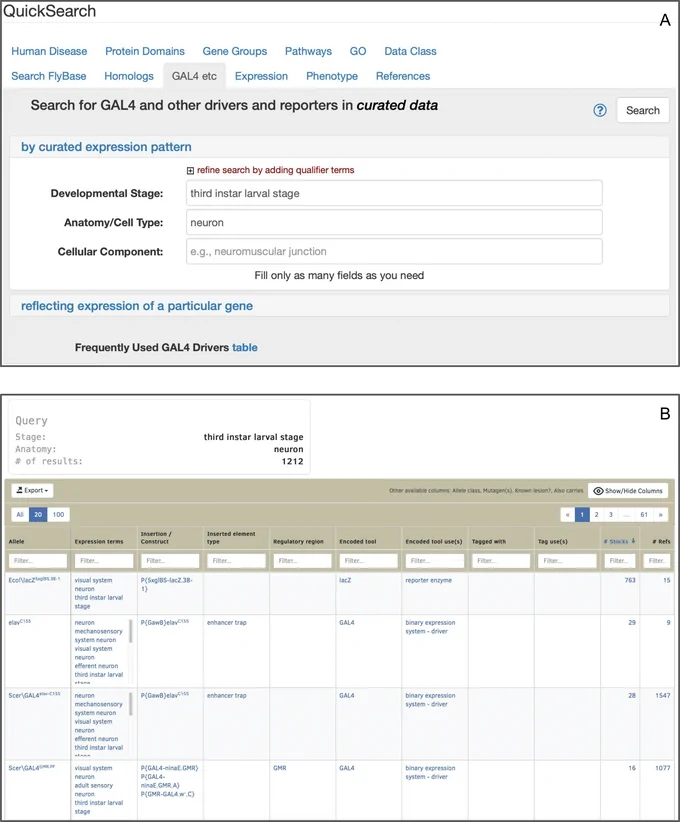
(**A**) The “GAL4 etc” tab of QuickSearch, with several criteria entered as search terms in the “by curated expression pattern” panel. (**B**) The integrated table of drivers and reporters resulting from that search.

This tab (Fig. 9) allows searching for GAL4 drivers, other binary system drivers (e.g., lexA and QF), and non‐binary reporters (e.g., lacZ or GFP) by temporal–spatial expression patterns, as curated from the literature (Thurmond et al., 2019). Expression data may be searched by developmental stage, anatomy/cell type, or subcellular location, or any combination of these. Alternatively, the lower panel of the “GAL4 etc” tab can be used to search for drivers/reporters that reflect the expression pattern of a specific gene (Larkin et al., 2021). This tab also provides access to an interactive table of “Frequently Used GAL4 Drivers” by clicking on the relevant text at the bottom of the tab. This table includes the most ordered GAL4 stocks from the Bloomington Drosophila Stock Center and drivers that have been curated to more than 20 publications (Larkin et al., 2021).

### Necessary Resources

#### Hardware

Computer or other device with access to the internet

##### Software

An up‐to‐date web browser, such as Firefox, Chrome, or Safari, with JavaScript and cookies enabled

1 Click on the GAL4 etc tab of QuickSearch (www.flybase.org). Choose whether to search for drivers/reporters by their curated expression pattern (upper panel) or by the gene whose expression they reflect (lower panel).
  These two search options cannot be combined, and only one may be chosen.

#### Search by curated expression pattern

2 If necessary, click on the “by curated expression pattern” text to expand this section (Fig. 9A).
3 Enter a term into the “Developmental Stage,” “Anatomy/Cell Type,” or “Cellular Component” field.
  Valid entries are terms or identifiers from the fly developmental stage, fly anatomy, or the GO cellular component ontologies, respectively. Valid terms that match the entered text will start to appear when typing and can be clicked to populate the field. Note that the autocomplete function only lists terms for which annotations exist. The search is case‐sensitive; wildcards (*) are not accepted.
  Note that searches will return hits matching the selected ontology term or any of its child terms. For example, searching with “imaginal disc” will return drivers/reporters annotated with that exact term as well as terms with an is_a relationship (e.g., wing disc and eye disc) or a part_of relationship (e.g., imaginal disc posterior compartment, wing pouch) to “imaginal disc.”
4 If desired, click the “refine search by adding qualifier terms” text and enter a field‐appropriate “qualifier” term in the box that appears below the main search box.
  For example, the developmental stage can be qualified by specifying gender, whereas the anatomy and subcellular fields can be qualified by a term adding detail about spatial distribution (e.g., “dorsal”) or signal intensity (e.g., “faint”). Valid qualifier terms that match the entered text will start to appear when typing and can be clicked to populate the field; only those qualifiers associated with the term chosen in step 3 will appear. The search is case‐sensitive; wildcards (*) are not accepted.
5 If desired, repeat steps 1 and 2 for the other search field(s).
  The autocomplete function is coordinated between the terms entered in the primary search boxes; only terms that have been co‐annotated with those already filled will be offered.
  It is not necessary to fill all three primary search boxes, but multiple fields may be completed in any combination to add additional constraints to a query.
6 Click the “Search” button or press “enter.”
7 View the integrated table of drivers/reporters that exhibit an expression pattern matching the search criteria (Fig. 9B).
  This table shows the connection between alleles, insertions, constructs, and stocks; additionally, the “Expression terms” column lists the anatomy, stage, and/or cellular component term that triggered the search result. The table is customizable; columns can be shown/hidden, moved, filtered, and specified as sort criteria. Results can be narrowed to a specific sort of driver or reporter, such as GAL4 or lacZ, by using the filter in the “Encoded tool”column.
8 Click on an individual allele, construct, or insertion to view its report page.
  Detailed expression information is included within the “Expression Data” section of the report.

#### Search by gene

9 Click on the “reflecting expression of a particular gene” text to expand this section.
10 Enter a gene symbol or ID into the search box.
  Valid entries are FlyBase gene symbols or identifiers.
11 Click the “Search” button or press “enter.”
12 View the integrated table of drivers/reporters that exhibit an expression pattern matching the search criteria.
  This table shows the connection between alleles, insertions, constructs, and stocks; additionally, the “Expression terms” column lists the anatomy, stage, and/or cellular component term that triggered the search result. The table is customizable; columns can be shown/hidden, moved, filtered, and specified as sort criteria.
  Note that this search will only return drivers/reporters that have been specifically curated as reflecting the expression pattern of the specified gene; it will neither find drivers/reporters with an expression pattern similar to that of the specified gene nor drivers/reporters that have not yet been curated as reflecting expression of that gene.
13 Click on an individual allele, construct, or insertion to view its report page.
  Detailed expression information is included within the “Expression Data” section of the report.

## Basic Protocol 10 USING THE “PHENOTYPE” TAB OF QuickSearch

**Figure 10:**
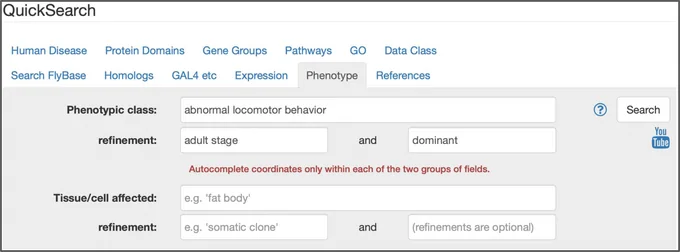
The “Phenotype” tab of QuickSearch, with several criteria entered as search terms within the upper “Phenotypic class” section.

This tab (Fig. 10) can be used to search for alleles (both classical and transgenic) associated with a specific class of phenotype, such as “lethal,” “sterile,” or “abnormal behavior,” or alleles that affect a certain anatomical or subcellular structure (Drysdale, 2001). These searches may be conducted in combination to retrieve alleles that match both criteria. There is also the option of restricting each search to a particular developmental stage.

### Necessary Resources

#### Hardware

Computer or other device with access to the internet

##### Software

An up‐to‐date web browser, such as Firefox, Chrome, or Safari, with JavaScript and cookies enabled

1 Click on the Phenotype tab of QuickSearch (www.flybase.org).
2 Enter a term in the “Phenotypic class” or the “Tissue/cell affected” fields.
  Valid entries are terms or identifiers from the phenotypic class, fly anatomy, or GO cellular component ontologies. Valid terms that match the entered text will start to appear when typing and can be clicked to populate the field. Note that the autocomplete function only lists terms for which annotations exist. The search is case‐sensitive; wildcards (*) are not accepted.
3 If desired, fill in one or both “refinement” boxes.
  These can be used to specify a stage of development or to specify qualifiers such as “somatic clone,” “dominant,” or “heat sensitive.” Valid qualifier terms that match the entered text will start to appear when typing and can be clicked to populate the field; only those qualifiers associated with the term chosen in step 2 will appear. The search is case‐sensitive; wildcards (*) are not accepted.
4 If desired, repeat steps 1 and 2 for the other search field.
  It is not necessary to fill in both the “Phenotypic class” and “Tissue/cell affected,” but doing so will add an additional constraint to a query.
  Note the autocomplete function is not coordinated between the “Phenotypic class” and “Tissue/cell affected” fields.
5 Click the “Search” button or press “enter.”
6 View the hit list of alleles associated with phenotype(s) matching the search criteria.
  Results are listed in descending order of their relevance score.
7 Click on an individual allele to view its Allele Report or choose to perform another action on the hit list (see Support Protocol 1).
  Detailed phenotypic information is included within the “Phenotypic Data” section of the Allele Report. Ontology terms are listed in the “Phenotypic Class” and “Phenotype Manifest in” subsections, alongside any qualifiers and accessory or transacting alleles. The “Detailed Description” subsection may include a “free text” description of the phenotype with additional qualifications that are not captured adequately by ontology terms alone.

## Basic Protocol 11 USING THE “HUMAN DISEASE” TAB OF QuickSearch

**Figure 11:**
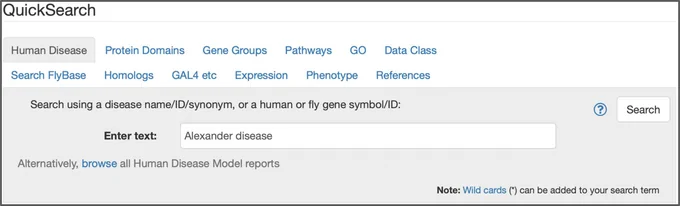
The “Human Disease” tab of QuickSearch, with the text “Alexander disease” entered as the search term.

The Human Disease tab (Fig. 11) allows access to data relevant to human disease models in *D. melanogaster*, which may be generated by expressing a human transgene in flies or by manipulating the fly ortholog(s) (Millburn et al., 2016). Diseases may be searched using terms from Online Mendelian Inheritance in Man (OMIM; Amberger et al., 2019) or the Disease Ontology (DO; Schriml et al., 2022); the DO is used in FlyBase to annotate classical and transgenic alleles that generate, ameliorate, or exacerbate a model of human disease. Genes and alleles annotated/associated with DO/OMIM terms can also be searched in this tab. Finally, this tab allows searching and browsing of Human Disease Model Reports, which are manually compiled reports that integrate all relevant data for diseases specifically modeled in *Drosophila*.

### Necessary Resources

#### Hardware

Computer or other device with access to the internet

##### Software

An up‐to‐date web browser, such as Firefox, Chrome, or Safari, with JavaScript and cookies enabled

1 Click on the Human Disease tab of QuickSearch (www.flybase.org).
2 Enter a term or an identifier corresponding to a disease, a human/fly gene, or an allele.
  Valid “disease” entries are disease names, synonyms, or identifiers, including those used in the FlyBase Disease Model Reports, the DO, and OMIM. Valid “human gene” entries are the gene symbols and IDs used by the HGNC (Seal et al., 2023) and OMIM (Amberger et al., 2019). Valid fly gene and allele entries are FlyBase symbols or identifiers. Valid entry terms will start to appear when typing and can be clicked to populate the field. The search is case‐insensitive and a wildcard (*) can be added to match partial terms.
  Alternatively, click on the “browse” link at the foot of the tab to see a page indexing all Human Disease Model Reports.
3 Click the “Search” button or press “enter.”
4 View the hit list of human disease models, DO terms, genes, and/or alleles matching the search criteria.
5 Click on an individual entry to view its corresponding report page or choose to perform another action on the hit list (see Support Protocol 1).
  Human Disease Model reports include an overview of the human disease, information on human/fly orthology relationships, human/fly alleles annotated with DO terms, potentially useful stocks, and lists of relevant references and external websites.
  DO term reports include a definition, synonyms, and a view of the tree displaying the hierarchical relationships between any parent and child terms. If applicable, the “Annotations” section includes links that will generate a new hit list of genes, alleles, or human disease models annotated with the given DO term or any of its child terms.
  Gene and Allele Reports list individual DO annotations with the “Human Disease Associations” section.

## Basic Protocol 12 USING THE “HOMOLOGS” TAB OF QuickSearch

**Figure 12:**
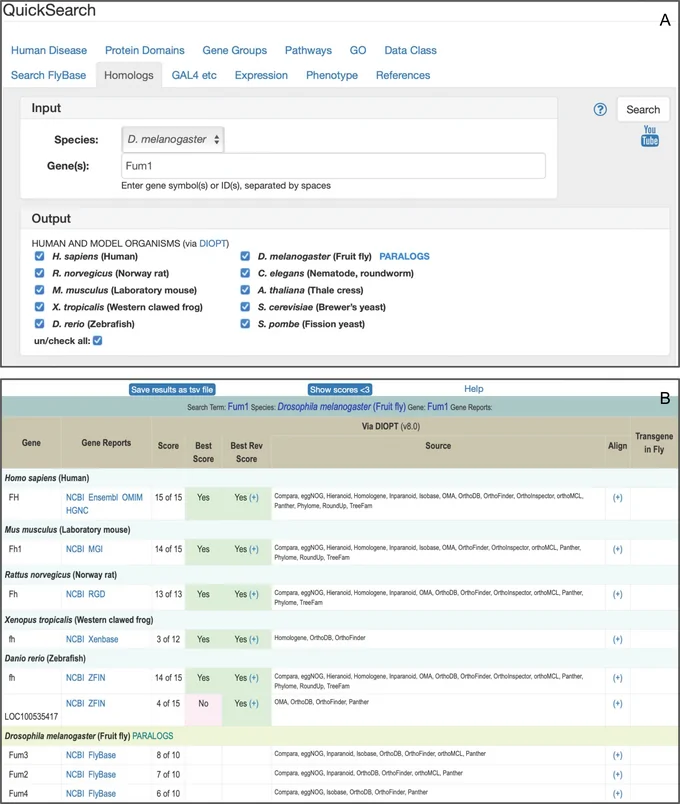
(**A**) The “Homologs” tab of QuickSearch, searching for orthologs and paralogs of the *Fum1* gene of *D. melanogaster*. (**B**) The results table from running that query.

This tab (Fig. 12) can be used to quickly search for orthologs of *D. melanogaster*, human, or other model organism genes, as well as paralogs of *D. melanogaster* genes, as provided by the DRSC Integrative Ortholog Prediction Tool (DIOPT) (Hu et al., 2011). DIOPT integrates predictions from many different algorithms based on sequence homology, phylogenetic trees, and functional similarity.

### Necessary Resources

#### Hardware

Computer or other device with access to the internet

##### Software

An up‐to‐date web browser, such as Firefox, Chrome, or Safari, with JavaScript and cookies enabled

1 Click on the Homologs tab of QuickSearch (www.flybase.org).
2 Select the input species from the “Species” drop‐down menu (Fig. 12A).
  The current input species are H. sapiens (human), R. norvegicus (Norway rat), M. musculus (laboratory mouse), X. tropicalis (Western clawed frog), D. rerio (zebrafish), D. melanogaster (fruit fly), C. elegans (nematode, roundworm), A. thaliana (thale cress), S. cerevisiae (brewer's yeast, and S. pombe (fission yeast).
3 Enter one or more gene symbols/identifiers in the adjacent “Gene(s)” box.
  Valid entries are gene symbols/IDs approved by the relevant species database or NCBI gene IDs. OMIM and Ensembl gene IDs are also accepted for human genes. Valid symbols will start to appear when typing and can be clicked to populate the field. Searches are case‐sensitive; wildcards (*) are not accepted. Multiple entries must be separated by a space character.
4 Select one or more output species using the checkboxes.
  The choice of output species is identical to the input species (see step 1). There is also the option of searching for paralogs if the input species is D. melanogaster.
5 Click the “Search” button or press “enter.”
6 View the results table (Fig. 12B).
  Results are presented for each individual input gene, sorted by descending “score.” The “score” is expressed as the number of algorithms supporting a given ortholog/paralog call compared to the total number of relevant algorithms for that call; the explicit list of supporting algorithms is also shown. Clicking the “Exclude scores <3” button at the top of the page filters out low‐scoring calls. The “Best score” and “Best Rev Score” columns indicate whether the given ortholog has the highest score for the query gene and whether the reciprocal relationship is also true.
7 If desired, click on the “+” symbol in the “Best Rev Score” column to perform a new homolog search with the given gene as the input.
  Conducting a reciprocal search gives a more complete view of complex homological relationships.
8 If desired, click on the “+” symbol in the “Align” column to view a pairwise alignment of the amino acid sequences.
  This links to an alignment page on the DIOPT website, which also tabulates the domains present in the two proteins and presents additional information on the individual algorithm scores.
9 If desired, click on a link in the “Gene Reports” or “Transgene in Fly” columns to view the corresponding Gene Report.
  Links are provided to report pages at model organism databases and NCBI; for human genes, additional links are provided to Ensembl and OMIM. Links in the “Transgene in Fly” column go to FlyBase Gene Reports for non‐Drosophila genes that have been expressed transgenically in flies.
10 If desired, download the visible results table as a TSV file.
  Click on the “Save results as tsv file” text at the top of the table to download all the results to a file in tab‐separated value format.

## Support Protocol 1 MANAGING FlyBase HIT LISTS

**Figure 13:**
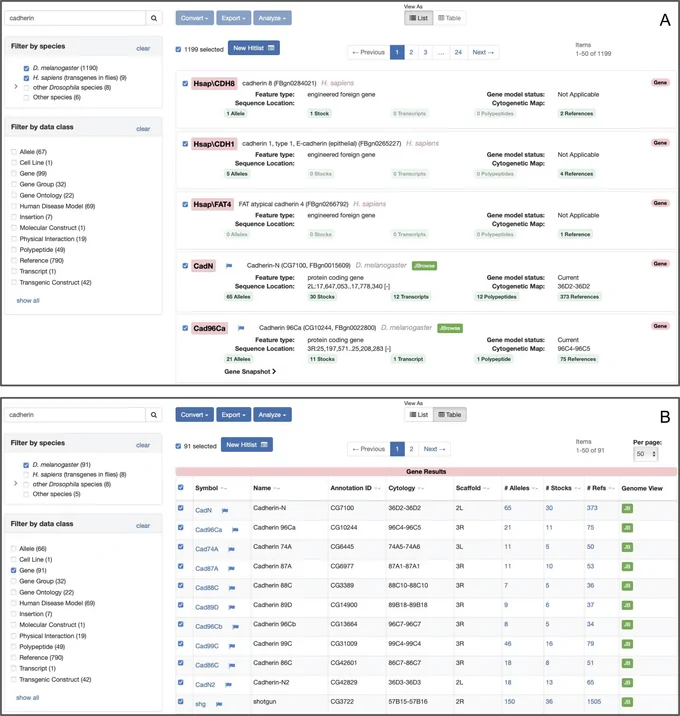
Hit lists resulting from searching with the text “cadherin” using the “Search FlyBase” tab of QuickSearch. (**A**) The default “List” view, showing hits from *H. sapiens* (transgenes in flies) and *D. melanogaster*. (**B**) The same hit list in “Table” view, after further filtering on *D. melanogaster* and the “genes” data class.

Most QuickSearch tabs return results as a “hit list” (Gramates et al., 2022; Jenkins et al., 2022; Thurmond et al., 2019; Fig. 13). In addition to presenting the data matching the search criteria, hit lists offer several powerful options to review, refine, convert, analyze, and export those data. The default view of a hit list is a “List” (Fig. 13A). This view can accommodate mixed data classes and presents each search result in a panel providing an extended overview of associated information. Alternatively, for results comprising a single data class, a hit list may be viewed as a “Table” by selecting that option under the “View as” text at the top of the page (Fig. 13B). In this case, search results are shown as separate rows of a table that has sortable columns showing associated data. Hit lists comprising a single data class may also be processed using the “Convert,” “Export,” and “Analyze” buttons. The following support protocol presents a guide to using these hit list features.

### Necessary Resources

#### Hardware

Computer or other device with access to the internet

##### Software

An up‐to‐date web browser, such as Firefox, Chrome, or Safari, with JavaScript and cookies enabled

1 Obtain a hit list by following one of the applicable basic protocols above or through another route within FlyBase (www.flybase.org).
2 If desired, (de)select individual entries in the hit list by clicking the checkbox next to the entry. All results can be (de)selected by clicking the checkbox at the top of the list of entries. This step may be performed at any point during filtering/processing.
  Selected entries can be used to generate a new hit list by clicking the “New Hitlist” button at the top of the page.
3 If desired/applicable, include or exclude data from different species by using the checkboxes in the “Filter by Species” box on the left of the page.
  Although the majority of FlyBase data pertain to D. melanogaster, reports also exist for genetic entities from other Drosophila species as well as non‐Drosophila species; the latter are required to accommodate data on heterologous expression, where a gene from a “foreign” species (e.g., GAL4, GFP, lacZ, or a human gene) has been expressed transgenically in D. melanogaster. The number in parentheses after each species or species group indicates the number of hits in that category.
4 If desired/applicable, include or exclude data from different data classes and ontologies by using the checkboxes in the “Filter by data class” box on the left of the page.
  The number in parentheses after each data class or ontology indicates the number of hits in that category.
  Note that selection of a single data class or ontology is a prerequisite for the subsequent (optional) steps.
5 If desired and if a hit list comprises a single data class, click on the “Convert” button at the top of the page to convert selected entries into a different applicable data class. Doing so generates a new hit list of the converted data class.
  For example, a list of genes may be converted into a list of alleles, transcripts, or stocks associated with those genes.
6 If desired and if a hit list comprises a single data class, click on the “Export” button at the top of the page to export selected entries into a different applicable FlyBase tool or as a downloadable file.
  For example, a list of genes may be exported to the Sequence Downloader, Ribbon Stack Viewer, Batch Download, QueryBuilder, or FeatureMapper tools.
  Any list may be downloaded as a simple text file of FlyBase IDs.
  Any list can be viewed as a “FlyBase cross‐referencing records table,” which tabulates all the FlyBase records with an internal cross‐reference to the submitted record(s).
7 If desired and if a hit list comprises a single data class, click on the “Analyze” button at the top of the page to analyze frequency values as histograms or view interaction maps of the selected entries, as applicable.
  For example, a list of genes may be analyzed for the frequency values of GO term annotations or chromosome arm locations, or displayed as a genetic/physical interaction network.

## COMMENTARY

### Background Information

Several improvements have been made to the interface and functionality of the QuickSearch tool since it was described in the previous protocol (Marygold et al., 2016). For example, two new tabs (“GAL4 etc” and “Pathways”) have been added to facilitate searching of driver/reporter lines and FlyBase signaling pathway reports (Larkin et al., 2021; Thurmond et al., 2019), whereas two other tabs [“Human Disease” and “Homologs” (previously “Orthologs”)] have undergone substantial revision (Gramates et al., 2022). Moreover, several aspects of QuickSearch were enhanced in conjunction with a general website upgrade in 2017, resulting in a refreshed interface and the transfer of all filtering options to the resulting hit list pages (Thurmond et al., 2019). Online documentation for QuickSearch has also been updated and links to YouTube tutorials have been added to tabs where available.

The QuickSearch tool allows rapid querying across all FlyBase data classes, ontologies, and major data annotations through a concise and intuitive interface. However, the input parameters and output processing options of QuickSearch are relatively limited, meaning that certain types of data (principally genomic data) and queries are not accessible via this tool. FlyBase provides an extensive set of additional tools to accommodate these use cases (St Pierre et al., 2014). For example, the QueryBuilder tool can be used to construct multi‐leg queries that search on a field‐by‐field basis, whereas three different RNA‐Seq tools offer the ability to search modENCODE RNA‐Seq data based on expression patterns or region‐specific expression levels. Other genomic data may be searched via BLAST, JBrowse, CytoSearch, or Feature Mapper. These tools, together with documentation on their use, are accessible via the “Tools” menu of the navigation bar present on every FlyBase webpage.

### Critical Parameters

It is important to use a QuickSearch tab that is suitable for the query. Most tabs search only a specific data class, ontology, or annotation and will result in an unproductive search if a term outside that class is entered. If in doubt or if a broad search is intended, then use the “Search FlyBase” tab. Note that orthological/paralogical and genomic data are outside the scope of this tab. In these cases, use the Homologs QuickSearch tab or a suitable genomic query tool (see Background Information), respectively.

Using a “valid” search term or ID will result in more accurate QuickSearch results. “Valid” in this sense means the “official” and current name, symbol, or ID of the entity (gene symbol, protein domain, ontology term etc.) in the source database. FlyBase is the source for *Drosophila* genetic symbols and terms from the phenotypic class, fly anatomy, and developmental stage ontologies, whereas different external databases are the sources for other terms (e.g., InterPro for protein domains, the GO Consortium for GO terms, or the HGNC for human gene symbols). To aid selection of a valid term, an autocompletion feature is active in all QuickSearch interfaces except for the general “Search FlyBase” tab. The autocomplete feature presents a list of matching terms that become more restricted as more characters are added to the search string. Searching with an invalid term will fail to autocomplete and is likely to generate either zero or inaccurate results. In this case, enter the invalid term into the “Search FlyBase” tab of QuickSearch (with the “Everything” option selected) and filter the results to identify the corresponding valid term/ID. Alternatively, the Vocabularies tool (also accessible from the homepage) may be used to search for valid ontology terms.

An asterisk (*) can be used as a wildcard in most QuickSearch tabs; exceptions are the Expression, GAL4 etc, Phenotype, and Homologs tabs. Wildcards can be added to the beginning, end, or middle of a search term to broaden the query. Flanking a search term with wildcards will return all phrases containing the search term.

Additional help documentation can be accessed online by clicking on the “?” icon within any individual QuickSearch tab.

### Troubleshooting

Common problems and possible solutions are shown in Table 4. Users should use the “Contact FlyBase” link present at the foot of every FlyBase webpage to request further assistance, report a bug, or suggest a new feature.

**Table 4:** Troubleshooting Common QuickSearch Problems

Problem	Possible cause	Solution
No results	‐ Search term is invalid ‐ Wrong tab was used	‐ Select a valid search term using autocomplete ‐ Use “Search FlyBase” tab
Too few results	‐ Search criteria are too restrictive	‐ Use the “Search FlyBase” tab ‐ Expand criteria by adding a wildcard ‐ Expand criteria by searching “everything” rather than “ID/Symbol/Name” (in Search FlyBase and Data Class tabs)
Too many results	‐ Search criteria are too broad	‐ Use the “Data Class” tab ‐ Restrict criteria by removing a wildcard ‐ Restrict criteria by searching “ID/Symbol/Name” rather than “everything” (in Search FlyBase and Data Class tabs) ‐ Exclude species and/or data classes/ontologies using filters on the resulting hit list
Missing/incorrect results	‐ Data have not yet been curated ‐ Data are not yet shown on website ‐ Curatorial or technical error	‐ Use the “Fast‐Track Your Paper” tool to check/prioritize curation of a paper ‐ Use the “Contact FlyBase” link to report issue to FlyBase

### Understanding Results

Results obtained using QuickSearch are an excellent starting place for further exploration. Nonetheless, there are some factors to bear in mind when interpreting these results, as outlined below.

Broad searches, such as those using the “Search FlyBase” tab, can often return undesired or false positive hits, whereas very restricted searches may result in relevant data being omitted from a hit list. Often, the most effective strategy is to begin a search with inclusive criteria (e.g., using the “Search FlyBase” tab) and then narrow these criteria (e.g., using the filters and analysis tools on a hit list, or repeating the query using a more restrictive QuickSearch tab) once the query is better defined and the underlying data are better understood.

The contextual reason for an item being returned in a search may not be obvious from the initial hit list. Therefore, before drawing any important conclusions, additional data associated with any hit of interest should be examined by clicking through to its report page in FlyBase. Additional context/clarification may be obtained by consulting the original data source, which is facilitated by the provision of clear attributions and links to references in FlyBase reports.

The FlyBase website is updated regularly (see the “Release Schedule” link in the “About” menu of the navigation bar), which means that the results of any QuickSearch query may change between releases. It is therefore important to note the FlyBase release number when conducting a query, as indicated in the header and footer of every FlyBase page. For example, the screenshots herein were taken from release FB2022_06.

### Time Considerations

It only takes a few seconds to run and obtain the results from a QuickSearch query, all other things being equal. However, the time required to process and understand the results will depend on the nature and purpose of the query. For example, it will only take a minute or two to find a specific allele, stock, or reference if the original search criteria are reasonably well defined. On the other hand, several minutes or hours may be required to perform a thorough analysis of all genes associated with a given GO term or all alleles associated with a specified phenotype.

### Author Contributions

**Steven J. Marygold**: visualization, writing original draft

### Conflict of Interest

The author declares no conflict of interest.

## Data Availability

QuickSearch and the entirety of FlyBase can be accessed freely at www.flybase.org.
